# Effect of Corn Starch as Stabilizer Particle in Combination with Egg White Proteins in Natural Rubber Latex Biofoams Produced by Microwave Foaming

**DOI:** 10.3390/polym17223057

**Published:** 2025-11-18

**Authors:** Clara Amezúa-Arranz, Leandra Oliveira Salmazo, Alberto López-Gil, Miguel-Ángel Rodríguez-Pérez

**Affiliations:** 1 Cellular Materials Laboratory (CellMat), Condensed Matter Physics Department, Faculty of Science, University of Valladolid, Campus Miguel Delibes, Paseo de Belén 7, 47011 Valladolid, Spain; leandra.oliveira@uva.es (L.O.S.); marrod@uva.es (M.-Á.R.-P.); 2 BioEcoUVA Research Institute on Bioeconomy, University of Valladolid, 47011 Valladolid, Spain; 3 CellMat Technologies S.L., Calle del Argón 1, 47012 Valladolid, Spain; a.lopez@cellmattechnologies.com

**Keywords:** low-density biofoams, open-cell structures, corn starch, egg white protein, sustainability

## Abstract

Current ecological and environmental concerns have led to a rapid increase in social interest in research and innovation in the field of sustainable plastics, which directly affects foamed plastic products. In this study, we present our contribution by investigating the effects of egg white protein and corn starch particles on open-cell biofoams produced from natural rubber latex in a two-step process based on an initial aeration that leads to a liquid foam precursor and its dehydration by microwave radiation. By incorporating corn starch and either replacing or maintaining the levels of egg white protein, two independent series of foams were examined. We observed how the reduction in egg white led to bigger and heterogeneous cells, although the density values were practically maintained around 100 kg/m^3^. In contrast, the formulations with corn starch at a fixed level of egg white protein created foams with homogeneous structures and smaller cells (≤120 µm). In addition, in terms of density, both series present values around 100 kg/m^3^ for the final solid foams, indicating that the addition of starch does not involve density increments. On the contrary, densities are still low, and the cellular structure homogeneity improves, confirming that starch is a very promising stabilizer bio-particle in the development of biofoams from liquids.

## 1. Introduction

Within the last few decades, plastics have become increasingly present in our daily life, mainly due to the fact that these materials are really versatile, with characteristics like low cost, thermal and electrical insulation, resistance to corrosion, and good mechanical properties relative to density, among others [1]. Global consumption of plastic is quickening, with over half of all the plastic ever manufactured having been produced since 2000, and this value is estimated to double by 2050 [2]. This massive rise in production leads to large CO_2_ generation, adding to the difficulties found in their end-of-life options, as most of them persist in landfills or the environment for generations.

The aforementioned ecological concerns are extrapolated to other kinds of materials based on plastic, like cellular polymers or polymer foams. A cellular material is a biphasic material in which a gas phase is dispersed into a solid or liquid phase named matrix [3] that could be based on metal, glass, ceramic, or polymer. The latter is especially present in a vast range of everyday applications such as impact absorption in automotive sector thermal or acoustic insulators, or mattresses and cushions in the comfort sector. Depending on the application or the kind of polymer, there exists a huge number of production processes to manufacture these cellular materials [4].

Particularly for polyurethane or latex foams, which are the main polymer foams in the comfort sector, an initial liquid foam is required to act as a precursor of the final solid expanded sample. In these cases, controlling the stability of those liquid foams is crucial to ensure a final stable solid foam and proper final physical properties, considering their final application [3]. To achieve this, a deep characterization of both kinds of foams, either the initial liquid foam or the final solid foam, is required to understand the influence of the different structural parameters on each other.

A liquid foam is a metastable system in which the following degeneration mechanisms could appear: drainage, coarsening, and coalescence [5]. To reduce degeneration mechanisms on these particular liquid foams, and overall drainage, the addition of several stabilizer additives is required such as surfactants, typically silicones or potassium oleate-based ones [6,7,8], to maintain bubbles within the liquid, and curing agents such as sulfur [9] to crosslink the polymeric matrix increasing the viscosity of the cell walls and vertexes promoting the stabilization of the cellular structure. Therefore, the formulations used are complex, and the addition of these additives leads to a final product not easily recyclable.

A common approach to improve the sustainability of foams in general, including those produced from liquid foams, is the use of either bio-based and compostable biopolymers or bio-additives in their formulations. Several works in the literature have been found, denoting the interest of academia and industry in this topic. For instance, gas dissolution foaming has been used with biopolymers such as poly(butylene adipate-co-terephthalate) (PBAT), poly(lactic acid) (PLA), or poly(butylene succinate) (PBS) [10,11]. Additionally, thermoplastic starch (TPS) has also been used in blends with some of the previous polymers to reduce their cost, tune their properties, and promote compostability [1,12,13,14,15]. TPS has also been used to produce biofoams by microwave radiation [16]. To further enhance sustainability, researchers have explored the incorporation of renewable additives and fillers such as fibers and agri-food subproducts. For example, orange peel-based particles have been introduced into TPS matrices during the casting process to develop films that can later be foamed, combining low-cost materials with improved environmental performance [15,17]. Focusing on cellular polymers derived from liquid foams, as in the case of polyurethane (PU) foams, finding alternatives has become crucial due to the toxicity of their reactants, particularly isocyanate. Increasingly, research efforts are directed towards the use of bio-based polyols derived from renewable sources, offering a greener approach to PU production [18]. Furthermore, new synthesis pathways are being explored, such as the reaction between carbonates and amines which leads to the attainment of non-isocyanate polyurethane (NIPU) foams, which eliminates the need for isocyanates [19,20].

In the case of natural rubber latex (NRL) foams, despite their bio nature, they are crosslinked either by sulfur or peroxides. There are previous studies aimed at making them more sustainable by incorporating renewable fillers [8,21,22,23,24]. Specifically, in a previous study [25], non-crosslinked rubber foams that were entirely bio-derived—as no sulfurs (crosslinking agent) or curing agents were used—were developed by using a more sustainable foaming process based on microwave radiation [26]. Instead, egg white protein (EW) was introduced as a stabilizing agent, further enhancing the sustainability of the material. Through this research, the effects of different amounts of EW on foams obtained from different latexes, and the resulting changes in the water content present in the polymer, were studied. These approaches enable low-density foams with stable, open-cell, and rather homogeneous structures to be obtained. EW was selected as an additive because these proteins both act as a surfactant, maintaining air bubbles based on the amphiphilic nature of the proteins, as well as a stabilizer, increasing the viscosity of the continuous matrix when proteins are denatured.

Despite its excellent foamability [27,28,29,30,31,32,33] and gelling ability [34], EW is an animal-derived product used mostly in food applications; its production process has led to diverse ethical and environmental problems, such as greenhouse gas emissions during chicken rearing for egg production, or the generation of human pathogens due to intensive chicken farming [35]. Due to these issues, there have been several attempts to find alternatives for these animal-based additives, with regard to their use in several applications and also in the development of novel biofoams based on natural rubber.

A promising alternative to egg white protein in the production of more sustainable rubber foams using microwave foaming is starch. Nowadays, starch is gaining interest due to its wide availability and low price, in addition to its other attributes, such as its biocompatibility, biodegradability, nontoxicity, and biosafety [36,37]. This biopolymer is obtained from cereals and tubers, and it is formed by two kinds of polysaccharides—a highly branched amylopectin and a linear amylose—which in their native form are arranged in semicrystalline granules [16,38]. Starch is used in the food sector as a thickening agent when it is gelatinized. The heating of dispersed starch granules in a liquid leads to the rupture of the intermolecular bonds of amylose and amylopectin, allowing hydrogen bonding to capture more water, and in consequence, the granules start to swell. At a determined temperature, the volume of the granules, and consequently, the viscosity of the blend, reaches a maximum. If the temperature continuously increases, the starch granules break down completely, and crystalline areas disappear, forming a gel that will boost the viscosity of the medium [39,40]. The fact that starch granules swell under heat in a water medium, increasing their viscosity, is a very promising aspect in the production of foams from aerated liquids, such as the rubber foams previously developed using latex and egg white protein. These liquid foams are heated and dehydrated using microwave radiation, and the presence of starch in the liquid interphase between bubbles may promote more stability.

The main objective of this work is to enhance the sustainable character of the NRL foams by introducing corn starch as a plant-based stabilizer. Two different series of foams are studied in this work: One series involves the replacement of EW with CS, allowing us to study the amount of protein that may be substituted without losing foam stability, and another series involves the addition of CS to a fixed amount of EW, so that we may compare the effects with those of previously studied EW formulations. The cellular structure of the foams obtained in the two stages of the process, as well as other aspects of the foams, were characterized in detail in order to find the possible synergies between both particles. The main novelty of this work resides in the introduction of CS as a plant-based stabilizer particle in combination with egg white proteins in a non-edible foam based on natural rubber latex intended for alternative applications such as packaging, cushioning, insulation, etc., in which bio-based foams show promise in terms of improved sustainability and circularity. The stabilization mechanisms of this plant-based particle and its possible interactions with egg white proteins are evaluated by means of detailed and quantifiable analysis of both the bubble and cellular structure in the two main stages of the process, aeration and dehydration by microwave radiation, and by observing how key parameters in the foams such as density, cell size, cell density, cell size distribution, etc., evolve from the liquid to the final solid foam. Such a detailed analysis of the cellular structure, for both the liquid and the solid foam, has not been carried out in previous scientific works as far as the author is aware, and could provide new insights into the analysis of solid foams produced from aerated liquids, as well as their aeration and foaming mechanisms.

## 2. Materials and Production Process

### 2.1. Materials

High-ammonia (HA) natural rubber latex (NRL), with 60 wt% dry rubber content and 40 wt% water, was supplied by LATEXA (Barcelona, Spain). The additives used in this work were egg white powder (EW) purchased from Harrison Sport Nutrition S.L. (HNS, Granada, Spain) and corn starch (CS) supplied by Roquette (Lestrem, France). Tap water is used to dilute latex to achieve the required water content.

### 2.2. Production Process of Natural Rubber Latex Foams

**Figure 1:**
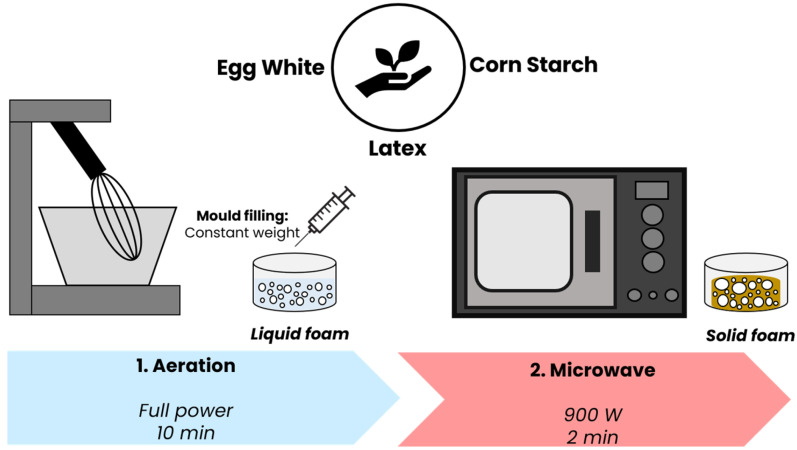
Lab route based on a two-step process carried out to obtain the samples under study.

The lab route carried out to obtain the samples developed through this research consisted of a two-step production process based on liquid aeration, followed by microwave heating [25] (Figure 1). The corresponding amounts of both kinds of particles, egg white powder and corn starch, were aerated with 100 phr of latex in a whipping machine (MUM 50123 StartLine, Bosch, Gerlingen, Germany) for ten minutes at full power (800 W). Then, the liquid foam obtained was immediately transferred into a 60 mL syringe to fill the cavity of a Teflon mold (diameter of 6.0 cm and height of 5.4 cm), maintaining this weight (10 g) in each experiment. Finally, the mold was introduced into a microwave (SHARP R-939, Sharp Electronics (Europe), Uxbridge, UK, with the following specifications: 40 L capacity, 2.45 GHz frequency, 900 W output power) in which the liquid foam was heated at 900 W for 2 or 3 min, depending on the series of materials (Table 1) to be completely dehydrated for the obtention of the final solid expanded sample.

In this study, all formulations were fabricated from latex with 70 phr of water. These samples were considered the most stable in a previous study [25] as they achieved the highest expansions with lower cell size increments after the microwave dehydration step. For all the samples of this work, the initial latex was diluted with tap water, so 50 phr of the initial latex (40 phr water content) was mixed with 50 phr of tap water to present a final water content of 70 phr.

Through this work, two different series of foams were obtained and characterized. In the first one, the total amount of stabilizer additives, a mixture of EW with CS, is maintained at 20 phr, but the relative amount between both is varied. The objective is to replace the animal-based additive (egg white powder) with a plant-based additive (corn starch). In the second series, the amount of EW is maintained at 20 phr, and the CS content is gradually increased from 10 to 40 phr, to evaluate the effect of this biostabilizer additive on the density, structure, and properties of the foam. In the first series of foams, the time spent in the microwave to achieve complete dehydration had to be increased from 2 min to 3 min.

**Figure 2:**
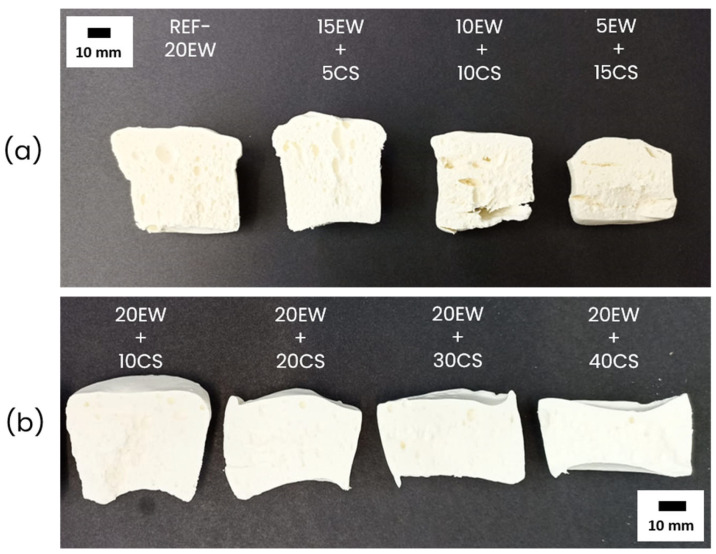
Solid NRLF series developed through this work: (**a**) Series 1 obtained by fixing 20 phr of additive, changing the ratios between particles, and (**b**) series 2 obtained by fixing 20 phr of EW, progressively increasing the amount of CS.

The cross-section of the final stable foams after microwave heating is shown in Figure 2; and both were analyzed separately. In terms of qualitative results for series 1 (Figure 2a), it is interesting to observe that by replacing EW with CS, stable foams are still obtained, even at very low contents of EW (5 phr). The internal cellular structure seems to become more heterogeneous as the CS content increases and the EW content decreases. Because of this, it is important to note that the sample obtained from 70WC latex and 5 phr of EW (70WC-5EW) in the previous work was not stable and collapsed [25]. Therefore, CS promotes the stabilization of the samples. Despite this, the presence of EW is important to achieve stable foams, as a foam with just 20 phr of CS collapsed. For series 2 (Figure 2b), increasing the amount of CS seems to compact the samples and restrict expansion, but with the naked eye, it could be observed that the homogeneity of the cellular structure is improved.

## 3. Characterization Techniques

This section contains a description of all the experimental techniques used to characterize the samples under study.

### 3.1. Density

The density values of liquid and solid foams were obtained geometrically by dividing the weight of each specimen using a precision balance (AT261 Mettler-Toledo balance, Mettler-Toledo, Barcelona, Spain) by its corresponding volume (ASTM standard D1622-08 [41]). The liquid foam density was calculated from the weight of the sample required to fill the Teflon mold (just after aeration, before the microwave stage) over the volume of the syringe displaced. Three measurements of each formulation were performed to ensure reproducibility. To obtain the density of solid foams, small cubic samples were cut from the middle of the final dehydrated foams. In this case, three replicates per formulation are measured to ensure reproducibility. Their geometric volume was determined by using a caliper of 0.01 mm resolution.

The relative density of the foams ($\rho_{r}$), for both liquids and solids, was obtained as the ratio between the density of the final foam ($\rho_{f}$) and that of the corresponding liquid or solid material that forms the matrix ($\rho_{s}$), as shown in Equation (1).

(1) $$\rho_{r}=\frac{\rho_{f}}{\rho_{s}}$$

For liquid samples, $\rho_{f}$ is the density of liquid foam measured just after whipping, and $\rho_{s}$ is the density calculated by the rule of mixtures of the initial liquid blend based on latex at 70 phr of water content and the corresponding amount of protein and starch. These values were obtained by considering the proportion of water/latex, and the following values: 1000 kg/m^3^ as the water density; the experimental density of the undiluted latex as 982 kg/m^3^; and the egg white powder and corn starch densities, measured using a gas pycnometer, were 1230 kg/m^3^ and 1494 kg/m^3^, respectively.

For solid samples, $\rho_{f}$ is the density of solid foam after the microwave step, and $\rho_{s}$ is the density of the solid matrix of the foam, which is a blend of the dry natural rubber present in the latex and the protein and starch added. In this case, the calculations were performed by the rule of mixtures, and the density of dry natural rubber was assumed to be 930 kg/m^3^ [9]. In addition, in this case, the amount of dry natural rubber was recalculated to consider the initial proportions of water diluted in the initial latex.

### 3.2. Cellular Structure

The cellular structure of the liquid and solid foamed samples were analyzed through optical micrograph analyses for liquid foams using a polarized light optical microscope (DM2500 M, Leica Microsystems, Wetzlar, Germany) with a digital camera (EC3, Leica Microsystems, Wetzlar, Germany) and a scanning electron microscope (FlexSEM 1000 VP-SEM, Hitachi High-Tech, Tokio, Japan) for solid foams. Optical microscopy images were taken two minutes after the aeration process—as this is the amount of time required to fill the mold and introduce it into the microwave—and ten minutes after, in order to study the temporal stability of the foams. Preparing the solid foam samples for SEM micrographs required cutting the foams in their middle zone and coating the surface with gold using a sputter coater (model SCD 004, BALZERS, Balzers, Liechtenstein). This enables the visualization of the growth plane (z) of the solid foam.

Image analysis software, ImageJ (version ImageJ 1.53t) [42], was needed to obtain the main structural parameters of both liquid and solid foams. The software performs a binarization process to isolate each bubble or cell from the rest.

To determine the size of each bubble/cell, the software selected its center and then measured its length from eight different angles, to finally calculate its average diameter The foam cell size is the average for all the cells considered in the image, and the average cell size in 3D (*ϕ*_3D_) was obtained by multiplying the 2D values by the correction factor 1.273 [42]. Up to 200 bubbles or cells were considered to ensure reproducibility.

The anisotropy ratio (R_y/x_) was calculated using Equation (2), which represents the ratio between the cell size/diameter of the cell in the expansion direction of the foam (z or D_1_) (*ϕ*_1_) and the cell size/diameter in one of the directions perpendicular to the latter (*ϕ*_2,3_).

(2) $$R_{y/x}=\frac{\phi_{1}}{\phi_{2,3}}$$

NSD or normalized standard deviation represents the homogeneity of the cellular structure. It is given by the ratio between the standard deviation of the cell size distribution and the average cell size of each foam sample (Equation (3)).

(3) $$NSD=\frac{SD}{\phi_{3D}}$$

In addition, cell density (N_v_) or the number of cells per unit volume of foam (cells/cm^3^), was determined according to Equation (4), and its expression is a function of the relative density (ρ_r_) and the cell size in 3D (*ϕ*_3D_) [42].

(4) $$N_{v}=\frac{6(1-\rho_{r})}{\pi {\left(\phi 3D\right)}^{3}}$$

### 3.3. Thermogravimetric Analysis (TGA)

TGA measurements were conducted on the solid foams under a nitrogen atmosphere using a Mettler Toledo TGA/SDTA 851, Mettler-Toledo, Barcelona, Spain. In the experiment, a piece of the solid foam (approximately 10 mg) was placed in an aluminum pan and heated using a temperature program from 50 °C to 850 °C at 20 °C/min.

### 3.4. Dehydration of Solid Samples

The percentage of dehydration in the solid samples was measured using Equation (5) to quantify water loss after the microwave process:

(5) $$\%\;Dehydration=\left[\frac{W_{l.f}-W_{s.f}}{W_{l.f}}\right]\times 100$$

where W_l.f_ is the weight of the liquid foam before microwave heating, and W_s.f_ is the weight of the solid foam after microwave heating.

This experimental value (% dehydration during the process) is compared with the theoretical water content of the initial blend (diluted latex with egg white protein and corn starch) before aeration. The 70 parts of water correspond to the sum of the phr of tap water introduced, added to the amount of water present in the latex (40% of the initial parts of latex). Finally, to calculate the percentage weight of water content, the phr of water previously obtained must be considered with respect to the total parts of the mixture (phr water content + phr latex solids + phr egg white protein + phr corn starch).

### 3.5. Mechanical Tests

Compression tests were performed by a universal testing machine (Instron model 5.500R6025, INSTRON, Barcelona, Spain) according to the standard ASTM D1621-00 [43] at room conditions of 23 ± 2 °C and 50 ± 10% of relative humidity, as indicated by ISO 291:2005 [44]. The displacement rate was [height/10] mm/min, and a load cell of 1 kN was used. The elastic modulus (E) was calculated from the slope of the linear region of the compression curves.

For clarity, it should be noted that the calculation of the elastic modulus in the cases where a linear region is not easily appreciated was performed by expanding the presuming linear region to identify the strain interval in which the curve behaves linearly. It is along these values that the linear regression was performed to obtain the modulus slope.

## 4. Results and Discussion

In this section, the results and characterization of liquid foams are going to be explained, and then the results and discussion related to solid foams will be examined. The two series of foams will be explained separately in both sections.

### 4.1. Liquid Foams

**Figure 3:**
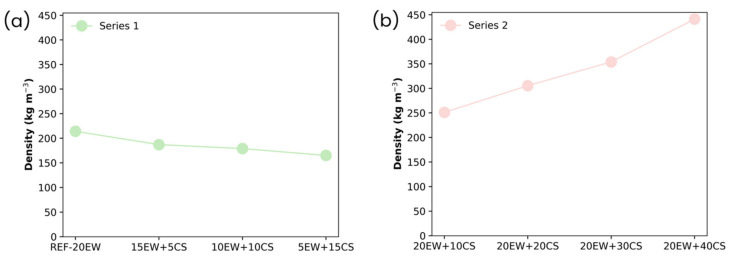
Density of liquid foams of both series of foams: (**a**) series 1, replacing EW with CS, and (**b**) series 2, adding CS to EW.

The purpose of this section is to gain insight into the events occurring during the aeration procedure, and the role played by the proteins and starch throughout this step. Therefore, an intensive characterization of liquid foams was carried out. The density values of all the formulations developed are shown in Figure 3.

In the foams obtained by fixing 20 phr of additive (Figure 3a), the density of the liquid foams does not vary significantly as EW is gradually replaced by CS. There is even a slight reduction (more aeration) when the dosage of CS is increased. On the contrary, in foams obtained by fixing 20 phr of EW (Figure 3b), their density trend differs from the first series, showing how the progressive increment of the amount of starch hinders the ability of the mixture to entrap air, as in this case, density increases progressively, reaching values more than double than those of the reference values without starch.

**Figure 4:**
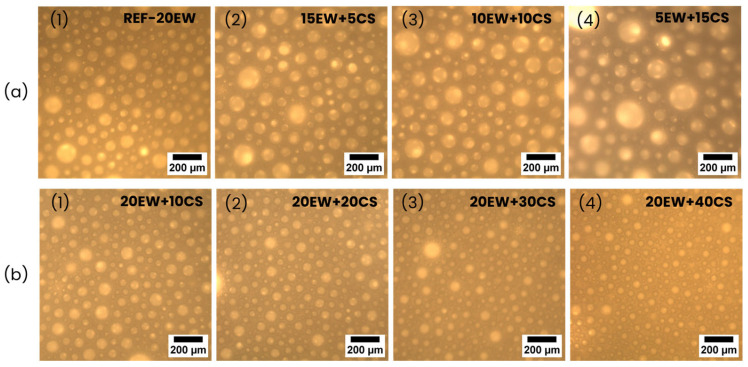
Optical microscopy images of (**a**) series 1, replacing EW by CS: (**a1**) REF-20EW, (**a2**) 15EW + 5CS, (**a3**) 10EW + 10CS, and (**a4**) 5EW + 15CS. For (**b**) series 2, adding CS to EW: (**b1**) 20EW + 10CS, (**b2**) 20EW + 20CS, (**b3**) 20EW + 30CS and (**b4**) 20EW + 40CS.

Figure 4 shows optical micrographs of the liquid foams of foams under study. In the reference sample (20 phr of EW, Figure 4a(1)), a heterogeneous bubble structure with diameters around 100 µm is observed. The replacement of EW by CS (series 1 of foams, see Figure 4a) appears to promote an increase in bubble size, but the heterogeneity is still high. The number of bubbles seems to become lower as EW content is reduced. The addition of CS to EW (series 2 of foams, see Figure 4b) seems to promote a reduction in the bubble size and maybe an increase in the number of bubbles trapped during the aeration process. Heterogeneity is still present, although it seems to be reduced in comparison with series 1.

**Figure 5:**
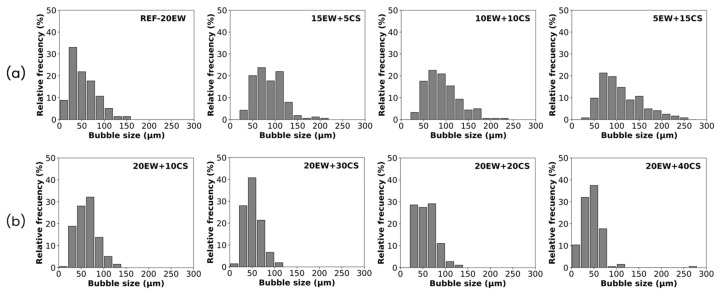
Bubble size distributions of the formulations under study: (**a**) series 1, replacing EW by CS, and (**b**) series 2, adding CS to EW.

A quantitative analysis is required to evaluate these possible trends in the bubble structure in more detail. This is observed first in the bubble size distributions obtained by image analysis (Figure 5) and then in the average parameters collected in Table 2.

The resulting histograms for series 1 (Figure 5a) show that bubble size distribution becomes wider when EW is gradually replaced by starch, indicating a progressive increase in the number of large bubbles ranging in values over 200 µm. This does not imply an increment in the heterogeneity of the structure, as NSD values of these samples are smaller than those of the reference values, staying rather constant.

In the case of bubble size distributions for series 2 (Figure 5b), all graphs tend to narrow and seem to have similar widths, although the presence of smaller bubbles is detected when CS is added. This could be an explanation for the larger values of the NSD coefficient, indicating an increase in the heterogeneity of samples.

In terms of the average cellular structure parameters, foams from series 1 present cellular structures characterized by mean bubble sizes that tend to progressively increase with the addition of CS. In consequence, the density of cells decreases while the amount of protein is replaced by CS. Finally, in terms of anisotropy, this value is around 1 for all the formulations, showing that bubble formation is isotropic, involving spherical bubbles in the liquid foam.

On the other hand, series 2 presents the highest values of relative density, and the average bubble size decreased in comparison with the reference, reaching values below 50 µm in some cases. In consequence, the density of bubbles gradually increases, although the change in these magnitudes is not abrupt. Finally, anisotropy occurs the same way as the previous series, and the values are around 1, as spherical bubbles are formed.

**Figure 6:**
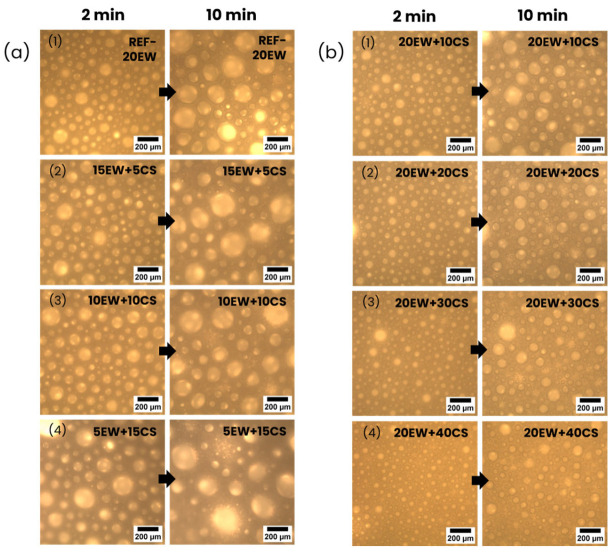
Optical microscopy images taken 2 min and 10 min after the aeration of (**a**) series 1, replacing EW by CS: (**a1**) REF-20EW, (**a2**) 15EW + 5CS, (**a3**) 10EW + 10CS, (**a4**) 5EW + 15CS, and after the aeration of (**b**) series 2, adding CS to EW: (**b1**) 20EW + 10CS, (**b2**) 20EW + 20CS, (**b3**) 20EW + 30CS and (**b4**) 20EW + 40CS.

The time stability of the liquid foams under study (Figure 6) has been analyzed to understand what occurs over time when these two additives are added. Optical microscopy images are taken 2 and 10 min after the sample’s aeration.

Regarding series 1 (Figure 6a), degeneration mechanisms occur as the bubbles grow, reducing their numbers over time. It seems that liquid foams with higher CS contents become more heterogeneous. Despite this, even at larger contents of CS (lower EW), the samples are still stable, as air bubbles are still present in the liquid foam. In the case of series 2 (Figure 6b), degeneration still occurs as bubbles grow through time, but is less intense than in the previous series. CS increments seems to avoid, up to a certain extent, the presence of degeneration mechanisms, as the bubble growth and diminution of bubble numbers are reduced.

**Figure 7:**
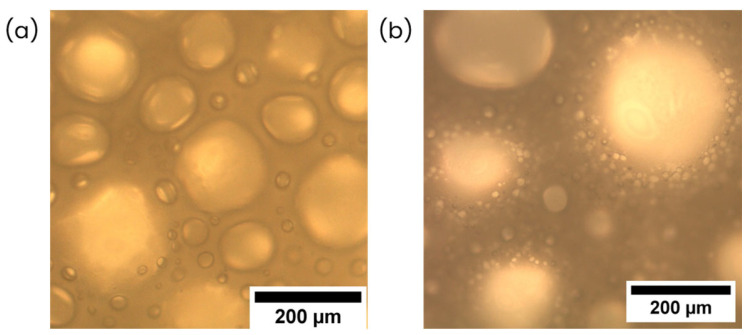
Optical microscopy images at high magnification of (**a**) reference—20EW and (**b**) 5EW + 15CS are used as an example in which the granules of CS can be seen as being displaced through the liquid phase of the foam.

In general terms, the key points that may be highlighted from the previous discussion are as follows: In the first series, where EW is replaced by CS, the air entrapped is maintained or even increases as density values decrease smoothly. This behavior is unexpected as the surfactant additive—the protein, which acts as a stabilizer in the interphase liquid–air—is replaced to introduce starch granules, which are not expected to act in the same way since they are hydrophilic particles and do not absorb at the air–water interphase [45]. Although these foams indicate higher levels of cellular degeneration as bubble size increases, and lower time stability as the number of bubbles decreases, liquid foams are still stable even in formulations with higher CS contents and lower amounts of EW (see Figure 6a(4), 5EW + 15CS). This may suggest that starch could have an additional stabilizing effect at this stage of the process (liquid foams), i.e., in the aeration, maintaining air bubbles. As proof of this effect, Figure 7 shows the microscopy image at high magnification compared to that of the reference with only protein, and the formulation with a higher CS content and lower EW is shown.

From Figure 7, it can be noted in Figure 7b the starch granules are located in the liquid phase around the existing bubbles. This suggests a possible trend of starch granules being placed near the interphase water–air surrounding air bubbles.

There are three possible hypotheses to explain this phenomenon. Firstly, the possibility that starch granules act as a “reinforcement” in the liquid phase, keeping the thickness of the film between bubbles, slowing the liquid drainage, and in consequence, the foam becomes more stable, reducing its density. These effects of starch particles in liquid foams have been found in previous studies [46,47]. Secondly, possible synergetic effects between EW and CS may also be occurring. There are several studies related to mixtures of protein and polysaccharides in aqueous systems [32,36,48,49,50,51,52] proving that polysaccharides may form complexes interacting with adsorbed proteins, improving their surface activity and increasing the rigidity at the interfaces [53]. The nature of this interaction may be covalent and non-covalent. Regarding the former case, proteins and polysaccharides can form permanent and irreversible complexes that exhibit high foaming stabilizing ability [54], whereas for the latter case, the interaction is led by secondary forces such as electrostatic forces, hydrostatic interaction, and hydrogen bonding [55,56]. This interaction has a significant influence on the structure and stability of dispersions and emulsions carried out in an aqueous system [57,58]. Finally, by decreasing the amount of proteins, their tendency to form aggregates during whipping, reducing their surfactant ability, is lowered [59]. In a nutshell, the present proteins act as surfactants, entrapping air bubbles, whereas starch granules are placed through the liquid phase, maintaining these bubbles and contributing to their stability.

Regarding the second series of foams, an increment in the liquid foam density is observed, although the number of bubbles entrapped in comparison with those of the reference sample remains practically constant or even increases. An explanation may lie in the increase in the initial liquid blend density due to a higher solid initial content, which could reduce its ability to entrap air bubbles. The possible stabilizer effect of starch granules previously commented on in liquid foams is also observed as the bubble sizes of this series are smaller, and also time stability seems to be enhanced, as the bubble losses are the lowest. There is existing literature about the effects of starch on an aqueous system with a fixed content of surfactant [46]. This study proved that there was a reduction in foamability with the amount of starch as the viscosity of the blend increased. The reduction in bubble size could be related to the large number of solids present in the liquid blend, which hinders the ability to entrap air. In fact, adding CS granules increases the thickness of the liquid foam walls/plateau borders [46]; so in consequence, the size of the bubbles will decrease, as will their amount.

The effect of the increment of CS on temporal stability could be crucial in the microwave stage, where they act as gelling or thickening agents when starch gelatinization is carried out [36].

### 4.2. Solid Foams

In this section, an in-depth characterization of the solid foams obtained from the second step of the production process is going to be carried out, to comprehend the events taking place during the microwave heating.

**Figure 8:**
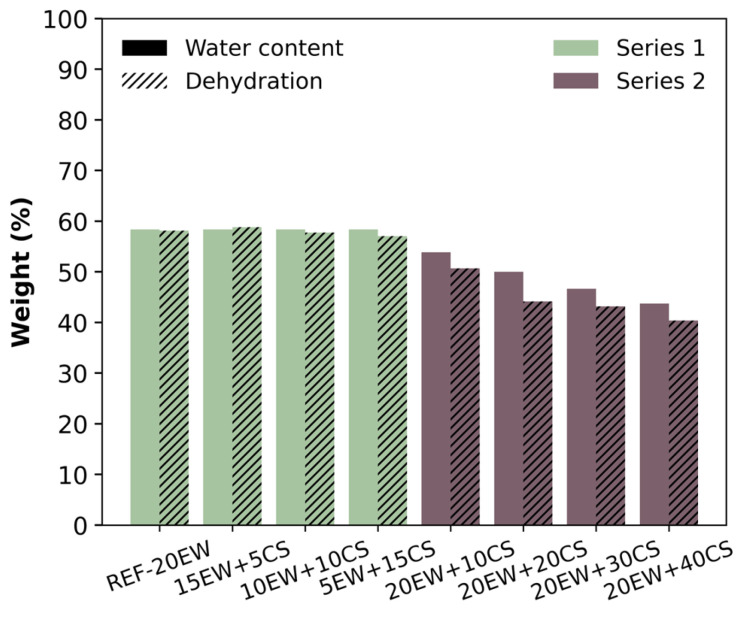
Graphic representation of theoretical water content (smooth bars) and dehydration values (striped bars) of all the foams obtained in this work.

Firstly, it is important to ensure that the solid foam samples are completely dried to ensure foam stability. Figure 8 collects the theoretical water content (smooth bars) and experimental dehydration values (striped bars) obtained following the procedure described in Section 3.4. Regarding both parameters, it can be deduced that the samples were completely dehydrated after microwave heating, as theoretical and experimental bars reach similar values. It is important to note that the time in the microwave for series 1 was increased from two to three minutes, to achieve complete dehydration.

**Figure 9:**
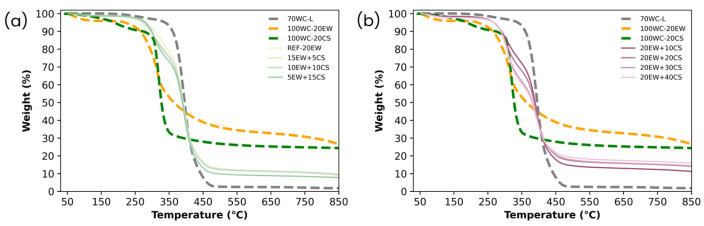
(**a**) TGA curves of series 1 (replacing EW with CS) and (**b**) series 2 (adding CS to EW). In both thermograms, foam obtained from 100 phr of latex with 70 phr of water content (70WC-L), foam obtained from 100 phr of tap water and 20 phr of protein (100WC-20EW), and foam obtained from 100 phr of tap water and 20 phr of corn starch (100WC-20CS) are used as a reference to compare the thermograms.

Figure 9 presents the TGA of the solid foams under study. Three references have been added to better comprehend the thermograms: A sample just produced with latex and no additives (70WC-L), another with water (no latex) and egg white protein (100WC-20EW), and the final reference obtained from water (no latex) and corn starch (100WC-20CS). All these samples help to explain the different weight drops linked to latex, egg white protein, and corn starch degradation. The references 70WC-L and 100WC-20CS did not lead to stable foams, but this did not affect this characterization.

For the foam produced just with latex, there is only one significant weight drop at 393.47 °C that is linked to the thermal degradation of isoprene [60]. The degradation of isoprene practically did not leave any residue. The foam obtained only with egg white powder has the main weight drop at 312 °C, linked to the thermal degradation of egg white proteins [61,62]. Finally, in the case of the foam with only corn starch, the first drop above 150 °C (189.70 °C) is related to the loss of absorbed or linked water, and the second and paramount drop around 319.15 °C is related to the degradation of starch [63]. The last stage finished with this drop, where the weight evolves from 90% to 24%, related to the formation of carbonaceous compounds [64].

Regarding the foams obtained, it could be considered that all the samples have two drops. The first one between 300 and 360 °C is associated with the degradation of both additives EW and CS, when in fact, both of them are overlapped in the thermograms of each foam. The second step, around 380 °C, is related to the decomposition of isoprene. As all the samples have the same content and type of latex, this drop is the same for all of them.

Now, the first drop previously mentioned is explained in detail. Focusing on series 1 (Figure 9a), the drop of the foam without starch (REF-20EW) has the same shape as the reference obtained only with protein (100WC-20EW), as both of them have the same amount of protein. While EW is replaced by CS, the shape of the curve changes to coincide with the reference obtained only with starch (100WC-20CS). For the foams of this series, this step is smaller than the drop that appears in the references due to the quantity of additives included in the foams being, in general, lower than in the references (samples obtained without latex). Regarding series 2 (Figure 9b), the trend followed by the first step of the thermogram of the samples is the same as the previous case, although this drop in trend is sharper due to the quantities of both additives being larger. In consequence, the amount of residue increases as well. The thermal degradation temperature of the foams is lower than the thermal degradation temperature of dehydrated latex (70WC-L) which is likely due to the presence of the stabilizer bio-additives whose thermal resistance is lower. However, it is interesting to note that the foams with both starch and proteins in both analyzed series present a higher thermal resistance than the reference foam with just proteins (100WC-20EW), which may indicate that the presence of isoprene in the foams delayed the thermal degradation of the additives in the foam and helps to improve the thermal resistance of the foams.

**Figure 10:**
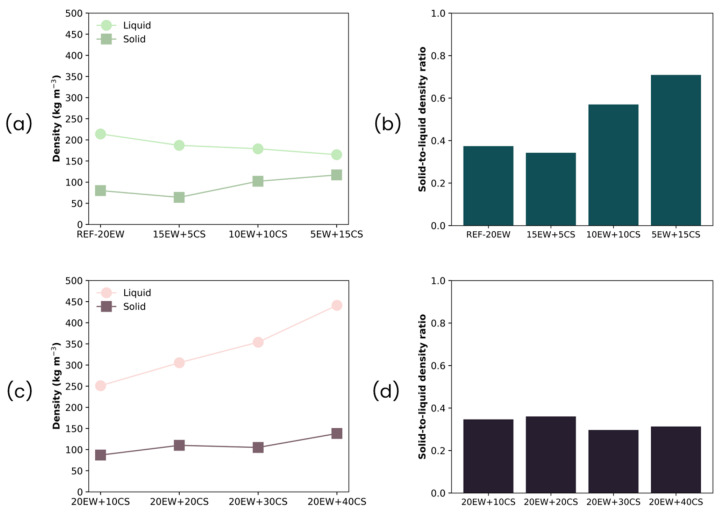
(**a**) Density of liquid and solid foams and (**b**) density ratio for series 1 (replacing EW with CS), (**c**) density of liquid and solid foams, and (**d**) density ratio for series 2 (adding CS to EW). Density values are obtained by means of three measurements for each formulation (n = 3).

The density of the liquid foams and their corresponding solid foams for both series is compared in Figure 10a,c. The density ratios for both series, shown in Figure 10b,d, is calculated as the density of the solid foam divided by the value of the liquid foam. Ratios lower than 1 indicate density reduction after the microwave step. All formulations developed through this work exhibit this trend.

Regarding density values, solid foams’ densities are always below those obtained for liquid foams. In series 1, the difference between liquid and solid foams is lower than in series 2 and tends to become similar as the amount of CS replacing EW increases. This is also observed by the increase in density ratio values, which are closer to 1, especially for the formulation with the lowest amount of protein (5EW + 15CS). Despite that, the solid foamed sample with the higher amount of starch is stable.

However, for series 2, the difference between solid and liquid foam densities is higher than in series 1, which is also confirmed by the low density ratios calculated. This difference becomes higher when the amount of starch increases due to the higher initial liquid foam densities obtained. This result suggests that during the microwave stage, the presence of higher contents of starch may promote foaming due to the steam generated, as evidenced by the lower ratio values below 0.4.

**Figure 11:**
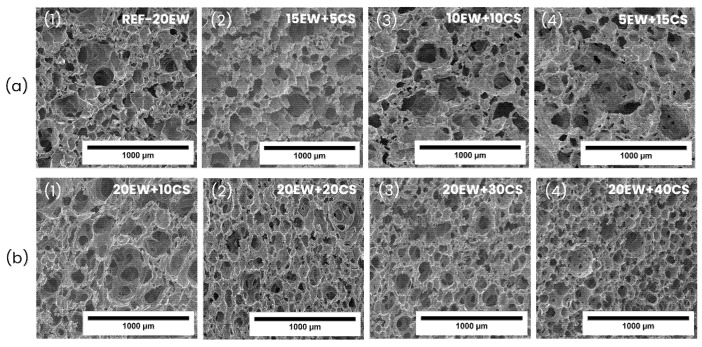
SEM micrographs of (**a**) series 1 (replacing EW by CS): (**a1**) REF-20EW, (**a2**) 15EW + 5CS, (**a3**) 10EW + 10CS, and (**a4**) 5EW + 15CS. SEM micrographs of (**b**) series 2 (adding CS to EW): (**b1**) 20EW + 10CS, (**b2**) 20EW + 20CS, (**b3**) 20EW + 30CS, and (**b4**) 20EW + 40CS.

The internal structure of the solid foams is also studied in detail through scanning electron microscopy (SEM). Micrographs of all the samples are shown in Figure 11. The micrographs of both series show partially open cellular structures in which many cells are interconnected by holes/ruptures of different sizes in cell walls. However, in general terms, two different kinds of structures are observed. For the first series (Figure 11a), the structure seems to be much more heterogeneous, as the samples present a huge variety of cell sizes and shapes overall, when the highest amounts of EW are replaced by CS. Whereas for the second series (Figure 11b), the samples are more homogeneous, and cell sizes tend to decrease as starch content increases, involving the formation of cells with rounded shapes.

**Figure 12:**
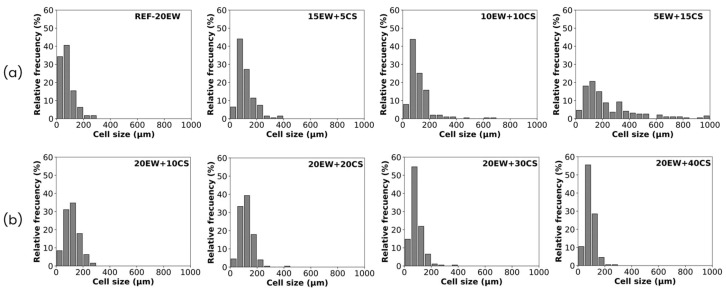
Cell size distributions of the formulations under study: (**a**) series 1 (replacing EW with CS) and (**b**) series 2 (adding CS to EW).

Quantitatively, cell size distributions and the mean structural parameters are presented in Figure 12 and Table 3. Concerning series 1 (Figure 12a), the cell size distributions become wider as proteins are replaced by starch, reaching values between 50 and 1000 µm for the last formulation, indicating an increment in the number of large cells when EW is replaced by CS. In addition, in terms of heterogeneity, the NSD values of these samples are the highest, being in most cases higher than 0.5, which is proof of the diversity of cell sizes and shapes observed in SEM micrographs.

Regarding series 2 (Figure 12b), all distributions tend to be narrower than in series 1, and the addition of CS leads to an increment in the number of cells with sizes lower than 100 μm. Moreover, the foams in series 2 are more homogeneous than in series 1, as their corresponding NSD coefficients are lower.

In terms of relative density, the values for this magnitude do not differ much, being below 0.100 for most samples in the two cases, although a slight increase in density is detected when replacing EW with CS, as in series 1, and when adding CS in series 2.

**Figure 13:**
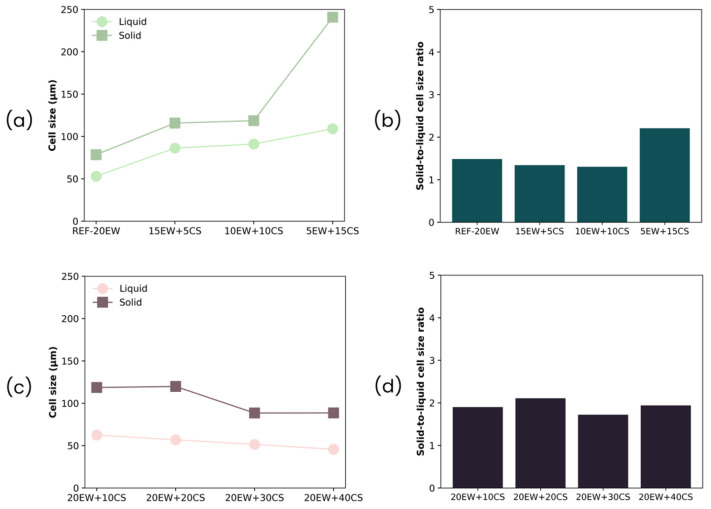
(**a**) Cell size of liquid and solid foams and (**b**) cell size ratio for series 1 (replacing EW with CS), (**c**) cell size of liquid and solid foams, and (**d**) cell size ratio for series 2 (adding CS to EW).

Regarding the average cell size, this parameter increases after the microwave stage in comparison with the average bubble size of liquid foams for both series of foams as observed in Figure 13. In the case of the foams belonging to the first series (Figure 13a,b), this trend is accentuated when higher amounts of EW are replaced by CS, as cell size varies from 78 to 240 µm. When EW is exchanged with CS, the liquid foam, which acts as a precursor, is less stable, as previously observed in the characterization of the liquid foams, promoting degeneration processes in the MW, which leads to higher cell sizes. This is proved by ratio values, which in all cases are above 1; specifically, in the case of the formulation with the lowest amount of protein (5EW + 15CS), its ratio increases almost double in comparison with the previous ratio.

Generally, in terms of anisotropy, the foams have ratios greater than one, showing a possible cell orientation during the microwave stage which is carried out inside a mold.

In series 1 of foams, when the particle acting as a surfactant (EW) is removed, the liquid foam exhibits a more degenerated structure with larger bubble sizes as previously observed in the characterization of the liquid foams. This directly affects the solid foam structure, as the cell size increases in foams with lower protein and higher starch content. However, the foams remain stable due to the presence of starch.

On the other hand, in the second series, even though cell sizes are superior to bubble sizes, it showed the opposite trend with regard to series 1 of foams, as cell sizes tend to decrease with the addition of starch. Although these foams initially show an increase when CS is added in comparison with the reference (REF-20EW), surging to 119 µm, the progressive increment of starch promotes a cell size reduction until it reaches similar values as the reference without starch (89 µm). As was explained before, this could be due to the increase in cell wall thickness produced by starch gelatinization and swelling, and due to an increase in viscosity of the liquid phase between bubble cells. Comparing the cell size ratio parameter in both liquid and solid foams (Figure 13c,d), cell sizes increase by almost double after the microwave process, but curiously, increasing the amount of CS does not significantly influence this difference in bubble cell size between liquid and solid foams, since the ratios tend to remain similar for all formulations. Regarding anisotropy, these samples presented slightly elongated cells (not spherical at all), affecting the values for this magnitude, which are the highest. In fact, sample 20EW + 20CS (Figure 11b(2)) has cells elongated to the growth direction related to a 1.18 value of anisotropy. In this case, preserving the EW content maintains the stability of the liquid foams, leading to more homogeneous structures. Moreover, the addition of starch improves stability, acting as a gelling agent that reinforces and thickens the cell walls.

**Figure 14:**
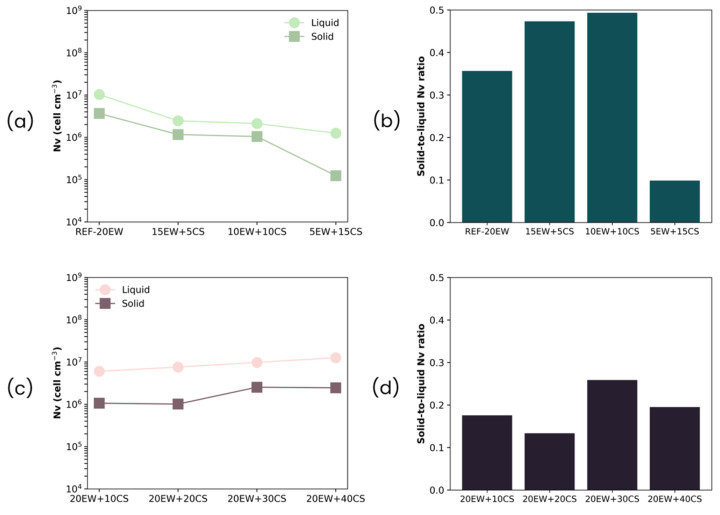
(**a**) Cell density of liquid and solid foams, (**b**) cell density ratio for series 1 (replacing EW with CS), (**c**) cell density of liquid and solid foams, and (**d**) cell density ratio series 2 (adding CS to EW).

Finally, regarding cell density (Table 3, Figure 14), for all the formulations, the ratio values are less than 1, which implies that the number of cells is reduced after the MW stage. This may be related to the increment in their size and the degeneration phenomena that may occur throughout the microwave process, leading to the rupture/union of the previous bubbles. Despite this effect, the degree of variation is not so high, as it maintains the order of magnitude in both types of foams (liquid and solid). Even so, the greatest changes, and consequently the highest ratios, can be observed for the first series (Figure 13b), where degeneration mechanisms predominate.

Both the density results and the cellular structure analysis reveal that starch also plays an important stabilizing role during the microwave step because it is gelatinized in the liquid phase during microwave heating, likely allowing, on the one hand, cell wall thickening, and on the other hand, an additional viscosity increment to the one occurring by protein unfolding.

In the first series of foams, this is evident because even at low protein contents, when starch is added, stable foams are still obtained with low final densities (≈100 kg/m^3^). However, the cellular structure clearly degenerates, leading to very large cell sizes, proving that proteins are also important in stabilizing the cellular structure in the microwave step.

On the other hand, in the second series of foams, the stabilization effect of starch is more significant, leading to very low final densities (like in the previous series ≈ 100 kg/m^3^) even though the initial liquid foam density is very high. The large density difference between the liquid foam and the solid foam at the highest starch content (40 phr) may be due to a second foaming step during microwave dehydration. New cells are generated in a liquid medium, which is much more stable due to the presence of starch. The cellular structures are also much more stable, and they become more homogeneous.

The behavior of both series can be explained by the behavior of both additives. In our last work [25], it was observed that the proteins not only act as a surfactant. In fact, when proteins are subjected to a thermal process, they unfold and create a three-dimensional gel network, which promotes stability to solid foam. In this new work, the samples also have corn starch. The temperature led to starch gelatinization, thickening cellular walls and boosting their viscosity, reducing degeneration mechanisms and stabilizing the sample. The behavior of each kind of series can be explained considering the previous explanations.

In the first series, reducing the amount of EW hindered the stability of the liquid foams that act as a precursor (their density decreases, temporal stability decreases, and their bubbles tend to larger sizes). Despite that, the addition of starch enables the maintenance of the structure, although the foams tend to be more heterogeneous. Another possible explanation for this tendency could be related to the following study [51]. In this work, they produced EW gels in which gelatinized starch granules are placed. They proved that by reducing EW and maintaining the concentration of CS, the resulting gel is less stiff. This could be an explanation as to why the foams present some large cells; the matrix is not tight/stiff enough to correctly retain the water steam generated and form homogeneous structures, and in consequence, expansion is reduced.

In the second series, all the formulations present a higher initial amount of protein (20 phr), which led to a stable liquid foam precursor. The addition of CS to the liquid blend will thicken cell walls, reducing cell size and increasing both cell density and homogeneity. The presence of the additive results in the reduction in degeneration mechanisms boosting the foam’s ability to retain water steam, and consequently, solid foams tend to have lower and similar density values. Moreover, CS in the presence of EW leads to an enhancement of the viscosity of the continuous matrix (Section S2—Supporting Information), which will promote the stability of the final foam, enabling the reduction in density while maintaining homogeneous cellular structure

**Figure 15:**
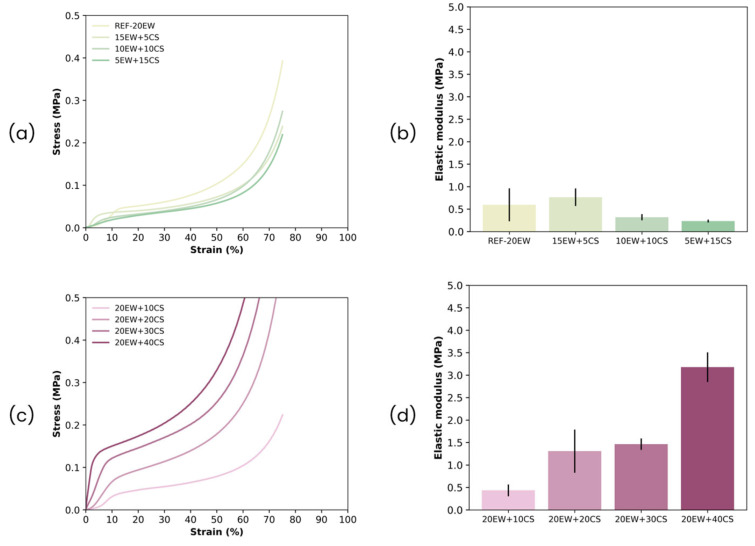
(**a**) Stress–strain curves and (**b**) elastic moduli for series 1; (**c**) stress–strain curves and (**d**) elastic moduli for series 2.

Finally, the last part of the study is related to gaining insight into the mechanical properties of the foams under study. Results obtained from the mechanical test of solid foams are displayed in Figure 15. Each series presents a different type of behavior, even when the relative densities of the samples do not differ much.

For series 1, as we replace EW with CS, the foams become less stiff (Figure 15a,b). This is verified from the resulting compression curves; at the same deformation values, lower stress values are obtained. Furthermore, modulus values also decrease. This phenomenon is interesting since the densities of these samples increase, so it would be expected that the modulus would also increase. These results may support the fact that replacing EW by CS promotes the formation of more flexible gels in the liquid foam and, as a result, more flexible cell walls and struts in the final solid foam, as previously found [51], such that the final solid foams are also less stiff (lower modulus are measured). The degeneration of the cellular structure of the samples in this series as starch gradually replaced proteins (cell size increases), could also directly affect the final mechanical properties, making the strength of the foam lower. And finally, this heterogeneity could also affect the final density of the samples, promoting a slight increment of this value.

For the second series, the starch stiffens (Figure 15c,d). As the amount of CS increases with fixed EW, the compression curves show how the stress gradually increases. In addition, modulus values increase abruptly, even reaching six times the reference (20EW + 40CS). This could be due to the reduction in the effect of the rubber present on the latex, as it has introduced the largest amounts of additives (EW and CS). So, in consequence, the flexible character of latex does not predominate as much.

**Figure 16:**
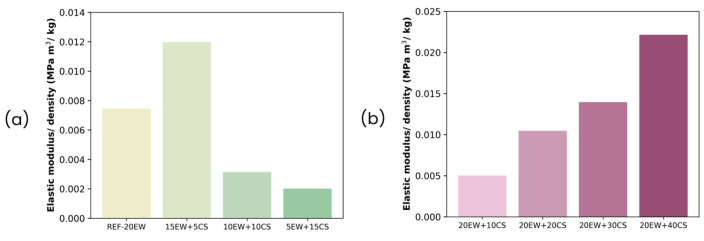
Relative elastic modulus for (**a**) series 1 and (**b**) series 2.

Figure 16 shows the specific modulus values (modulus divided by the foam density) [65] to exclude the effect of the latter, as the foams studied present different density values. As can be seen, the values for each formulation are not equal, proving that the mechanical behavior is a result of the cellular structure and the type of polymer matrix in the walls.

## 5. Conclusions

Through this work, we developed two series of rubber foams based on latex with 70 phr of water content and EW and CS as additives and gained insight into the stabilization mechanisms presented by these particles on both liquid and solid foams after the two steps of the production process.

By studying the two-foam series, we were able to determine the combined effects of both particles. On the one hand, by studying the first series of foams, replacing EW with CS leads to a more unstable liquid foam precursor, which gives rise to a solid foam prone to degeneration with more heterogeneous cellular structures, characterized by larger cell sizes (241 µm for 5EW + 15CS, in comparison to 78 µm of the reference without starch). However, despite these changes, the resulting solid foams remain stable, and the final density values achieved are still low (117 kg/m^3^ for 5EW + 15CS). On the other hand, from the second series, adding CS to the original EW content results in a stable liquid foam precursor, which led to a homogeneous solid foam with smaller and rounded cells (88 µm for 20EW + 30CS) due to wall thickening resulting from starch gelatinization. Despite the large amounts of additives presented in this series, a significant reduction in density occurs during the microwave step, achieving solid foams with densities around 100 kg/m^3^.

These results prove that the use of starch granules in foams produced from liquids, such as the latex-rubber foams produced in this work, could provide more stability not only during the dehydration step, or due to starch swelling, cell wall thickening and viscosity increment, but also during the liquid foam phase in which some possible synergies with egg white proteins could also help to provide surfactant-based stability to the bubble structure formed, which are also reflected in the final solid foams. This is a very unique result in the study of biofoams that are not used in food.

The applicability of these foams may lie in the fields of sustainable packaging, given their full bio-based composition, and for acoustic insulation, given their low density and open-cell structure. Currently, we are focusing on the production of large prototypes to perform full characterization of these properties, our main goal being to enhance sustainability in this area through the development of these bio-based foams.

## Figures and Tables

**Table 1:** Formulations of the NRLF developed through this work.

Series	Sample	Egg White Protein (phr) ^a^	Corn Starch (phr) ^a^
Series 1 REPLACING EW BY CS: 20 phr stabilizer additives (sum of EW and CS)	REF-20	20	0
	15EW + 5CS	15	5
	10EW + 10CS	10	10
	5EW + 15CS	5	15
Series 2 ADDING CS TO EW: 20 phr EW + different contents of CS	20EW + 10CS	20	10
	20EW + 20CS	20	20
	20EW + 30CS	20	30
	20EW + 40CS	20	40

^a^ Parts per hundred of NRL.

**Table 2:** Density and average structural parameters of liquid foams developed through this work.

Sample	ρ (kg/m^3^)	ρ_r_	Φ_3D_ (µm)	NSD	R_y/x_	N_v_ × 10^6^ (cells/cm^3^)
REF-20	214 ± 13	0.206	53 ± 29	0.56	1.02	10.26
15EW + 5CS	187 ± 11	0.177	86 ± 33	0.38	0.98	2.45
10EW + 10CS	179 ± 16	0.167	91 ± 36	0.40	1.00	2.12
5EW + 15CS	165 ± 18	0.151	109 ± 47	0.43	0.98	1.25
20EW + 10CS	251 ± 15	0.231	62 ± 24	0.38	0.98	6.04
20EW + 20CS	305 ± 17	0.271	57 ± 23	0.40	1.03	7.57
20EW + 30CS	354 ± 14	0.305	51 ± 19	0.37	0.97	9.80
20EW + 40CS	441 ± 25	0.372	46 ± 24	0.53	0.99	12.60

**Table 3:** Density and average structural parameters of solid foams obtained through this work.

Sample	ρ (kg/m^3^)	ρ_r_	Φ_3D_ (µm)	NSD	R_y/x_	N_v_ × 10^6^ (cells/cm^3^)
REF-20	80 ± 5	0.075	78 ± 40	0.51	1.12	3.66
15EW + 5CS	64 ± 2	0.058	116 ± 50	0.43	1.21	1.16
10EW + 10CS	102 ± 11	0.089	119 ± 66	0.56	1.06	1.04
5EW + 15CS	117 ± 20	0.099	241 ± 157	0.65	1.00	0.12
20EW + 10CS	87 ± 6	0.075	119 ± 41	0.35	1.02	1.06
20EW + 20CS	110 ± 3	0.090	120 ± 40	0.34	1.18	1.01
20EW + 30CS	105 ± 5	0.083	88 ± 36	0.40	1.30	2.53
20EW + 40CS	138 ± 18	0.106	89 ± 29	0.33	1.26	2.46

## Data Availability

Data is contained within the article.

## Supplementary Materials

The following supporting information can be downloaded at: https://www.mdpi.com/article/10.3390/polym17223057/s1, Figure S1: NRL foam (10EW+10CS) dehydrated by (a) a conventional oven and (b) a microwave. Figure S2: RVA curves of (a) 10 phr of CS (10CS) and the following protein/starch (phr/phr) mixtures: (b) 5EW+7.5CS, (c) 5EW+10CS, and (d) 5EW+12.5CS. References [66,67,68,69,70,71,72] are cited in the supplementary materials.
