# Supplementary material for: Effect of Corn Starch as Stabilizer Particle in Combination with Egg White Proteins in Natural Rubber Latex Biofoams Produced by Microwave Foaming
Source: Polymers (Basel). 2025 Nov 18;17(22):3057. doi: 10.3390/polym17223057 (PMC12656337; doi:10.3390/polym17223057)
Supplement: Supplementary file 1 [file polymers-17-03057-s001.zip › polymers-3867087-supplementary.pdf]

SUPPORTING INFORMATION

1. *Differences between convention heating and microwave heating*

The next lines explain the reasoning behind the selection of microwave radiation as a heating method. **Figure S1** presents a comparison between one of the foam formulations (10EW + 10CS) obtained following the procedure described in Section 2.2, with the only difference of the heating method employed in the second step: conventional heating (**Figure S1.a**) and microwave heating (**Figure S1.b**), as temperature and time parameters in both cases are the same. For conventional heating, the temperature selected was 100˚C, as this is the one expected to achieve during microwave dehydration. The duration of the second step was set in 3 minutes, as it is the time required to achieve complete dehydration via microwave radiation for the series that belongs this foam formulation. The selection of the formulation has been arbitrary; it does not affect the behavior of the final foam.

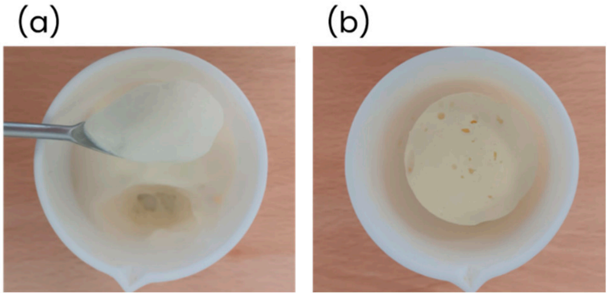

Figure S1. NRL foam (10EW+10CS) dehydrated by (a) a conventional oven and (b) a microwave.

As can be observed in **Figure S1. a,** the foam is not dehydrated, and the liquid foam can still be collected with a spoon. In contrast, in **Figure S1.b**, the sample exposed to microwave radiation for the same time results in a stable solid foam.

This behavior is related to the heat transfer mechanism. In the first case, a convection oven relies on surface heating, transferring heat from the edge to the inside, thus generating a temperature gradient in that direction. The image demonstrates that the heating time was not enough to achieve uniform heating throughout the sample, sufficient to produce water vapor for cell formation and to induce starch gelatinization and protein denaturation, both necessary to stabilize the solid foam. On the other hand, the microwave provides volumetric heating, where heat is generated from the inside out due to the vibration of water molecules. This type of heat transfer is uniform throughout the sample and significantly faster, allowing the formation of a stable solid foam

2. *RVA measurements*

2.1 Device and method

Viscosity measurements were performed on different ratios of the additives by using a rapid viscoanalyzer (RVA 4500, PerkinElmer, Hägersten, Sweden). This mixture was initially held at 50 ˚C for 60 s, then the temperature was gradually increased at a rate of 6◦C/min. After reaching 95 ˚C, the temperature was maintained for 5 min, followed by a decrease at the same rate of 6 ˚C/min. For the final 2 min of the procedure, the temperature was stabilized at 50◦C. Throughout this

process, the viscosity of the samples was continuously monitored at varying temperatures. The rate of the viscosimeter was set at 160 rpm. The experiments were conducted in water since latex could not be analysed under these conditions due to its nature and unsuitable viscosity range for the equipment.

2.2 Results

Figure S2 is presented as proof of the increment of the viscosity of the system when CS is found in the presence of EW; several RVA measurements were performed, including several viscosity profiles of starch and different ratios of protein-starch mixtures. Due to device specifications, avoiding measurements at high liquid viscosities, the selected amount of additives was lower than the one that is under study.

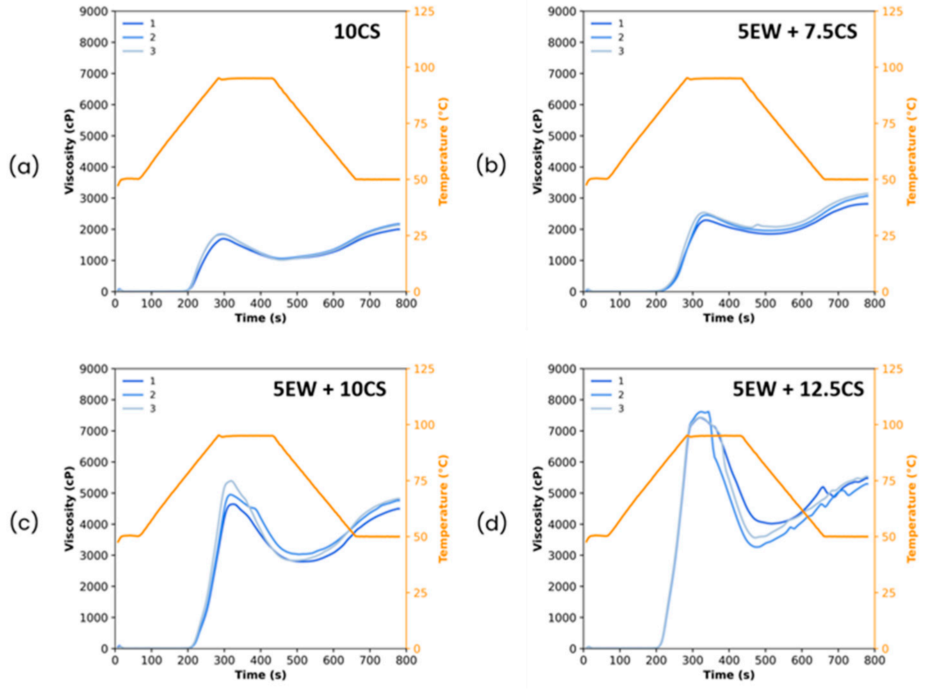

Figure S2. RVA curves of (a) 10 phr of CS (10CS) and the following protein/starch (phr/phr) mixtures: (b) 5EW+7.5CS, (c) 5EW+10CS, and (d) 5EW+12.5CS.

All the curves present the profile of starch gelatinization [1–3]. When the applied temperature exceeds the one for starch gelatinization, the viscosity starts to increase (pasting temperature) due to the swelling and partial rupture of the granules. It seems that the gelatinization process starts at the same temperature (200 ˚C) for all formulations.

During the initial heating stage, there is an increment in the system viscosity until it reaches a maximum (peak viscosity) due to the swelling of the starch granules induced by hydrothermal effects. We can observe how the presence of the protein at the same amount of starch increases this value (compare **Figure S2.a,c**). Besides, the more starch in the formulation, the greater increment of the viscosity through this step (compare **Figure S2.b,c,d**), as more granules are subjected to gelatinization [4]. The subsequent temperature holding leads to a decay in this parameter due to a gradual disruption of swollen starch granules by shear effects [5].

Then, at the final cooling stages, viscosity increases again due to the re-crystallization of starch or retrogradation until it reaches the final viscosity. As it has been mentioned at the end of Section 4.1, some of the molecular interactions between starch and protein can occur in terms of

electrostatic forces, hydrogen bonding, and hydrophilic interactions. This affects starch retrogradation as hydrophobic groups hinder the amylose release and reassociation, while hydrophilic groups impact water or molecular mobility [6].

Particularly, there exist studies that note how the high solubility of egg albumen (the predominant protein in egg white) enables it to retain less water and allows starch to swell more within the blend, promoting gelatinization. The improved dispersion of starch inhibited retrogradation by enabling greater interaction with water molecules. Water-soluble proteins, such as albumen, influenced water mobility between starch granules/matrix, reducing retrogradation rate [6–8].

Regarding the graphs of the mixtures, it can be shown an increase in the setback related to starch retrogradation. This trend could be due to the increment of the final amount of polysaccharide, which increases the number of chains of amylose and amylopectin susceptible to reassembly. Despite the effect of ovalbumin on starch retrogradation previously mentioned, the impact of protein-starch interactions on retrogradation are highly dependent of the protein type (source, amino acid sequence, structre, etc.), starch type (source, amylose/amylopectin ratio, etc.), protein/starch/water ratio, and processing conditions [6]; thus proteins, can either promote or reduce retrogradation depending on those specifications.

References

[1] M. Schirmer, M. Jekle, T. Becker, Starch gelatinization and its complexity for analysis, Starch - Stärke. 67 (2015) 30–41. https://doi.org/10.1002/star.201400071.

[2] X. Zhang, X. Lin, B. Xu, Morphological, physicochemical, and pasting properties of pre-gelatinized starch prepared by high-pressure homogenizer: A comparative study on starches from different resources, Food Research International. 197 (2024) 115294. https://doi.org/10.1016/j.foodres.2024.115294.

[3] D. Hu, G. Yang, Y. Tian, M. Li, L. Fan, R. Li, S. Wang, Effect of radio frequency heating on structure and physicochemical properties of protein and starch based on gelatinization degree of rice flour, Food Research International. 218 (2025) 116902. https://doi.org/10.1016/j.foodres.2025.116902.

[4] L.M. Ignatzy, K. Kern, I.S. Muranyi, T. Alpers, T. Becker, S. Gola, U. Schweiggert-Weisz, Thermal, rheological, and microstructural characterization of composite gels from fava bean protein and pea starch, Food Hydrocolloids. 172 (2025) 111883. https://doi.org/10.1016/j.foodhyd.2025.111883.

[5] J. Wang, S. Zhao, G. Min, D. Qiao, B. Zhang, M. Niu, C. Jia, Y. Xu, Q. Lin, Starch-protein interplay varies the multi-scale structures of starch undergoing thermal processing, International Journal of Biological Macromolecules. 175 (2021) 179–187. https://doi.org/10.1016/j.ijbiomac.2021.02.020.

[6] G. Scott, J.M. Awika, Effect of protein–starch interactions on starch retrogradation and implications for food product quality, Comprehensive Reviews in Food Science and Food Safety. 22 (2023) 2081–2111. https://doi.org/10.1111/1541-4337.13141.

[7] E. Waziiroh, D. Bender, P.L. Fuhrmann, R. Schoenlechner, Effect of non-covalent interactions on gluten-free batter stability and bread properties, LWT - Food Science and

Technology. 215 (2025) 117263. https://doi.org/10.1016/j.lwt.2024.117263.

[8] R. Crockett, P. Ie, Y. Vodovotz, Effects of soy protein isolate and egg white solids on the physicochemical properties of gluten-free bread, Food Chemistry. 129 (2011) 84–91. https://doi.org/10.1016/j.foodchem.2011.04.030.
